# Defining the need for public health control of scabies in Solomon Islands

**DOI:** 10.1371/journal.pntd.0009142

**Published:** 2021-02-22

**Authors:** Susanna J. Lake, Daniel Engelman, Oliver Sokana, Titus Nasi, Dickson Boara, Anneke C. Grobler, Millicent H. Osti, Ross Andrews, Michael Marks, Margot J. Whitfeld, Lucia Romani, John M. Kaldor, Andrew C. Steer

**Affiliations:** 1 Tropical Diseases Research Group, Murdoch Children’s Research Institute, Melbourne, Australia; 2 Department of Paediatrics, University of Melbourne, Melbourne, Australia; 3 Melbourne Children’s Global Health, Melbourne, Australia; 4 Ministry of Health and Medical Services, Honiara Solomon Islands; 5 Clinical Epidemiology and Biostatistics Unit, Murdoch Children’s Research Institute, Melbourne, Australia; 6 Australian National University, Canberra, Australia; 7 London School of Hygiene and Tropical Medicine, London, United Kingdom; 8 Hospital for Tropical Diseases, London, United Kingdom; 9 St Vincent’s Hospital, University of New South Wales, Sydney, Australia; 10 Kirby Institute, University of New South Wales, Sydney, Australia; International Foundation for Dermatology, London, United Kingdom, UNITED KINGDOM

## Abstract

Pacific Island countries have a high burden of scabies and impetigo. Understanding of the epidemiology of these diseases is needed to target public health interventions such as mass drug administration (MDA). The aim of this study is to determine the prevalence of scabies and impetigo in Solomon Islands as well as the relationship between them and their distribution. We conducted a prevalence study in 20 villages in Western Province in Solomon Islands. All residents of the village were eligible to participate. Nurses conducted clinical assessments including history features and skin examination. Diagnosis of scabies was made using the 2020 International Alliance for the Control of Scabies diagnostic criteria. Assessments were completed on 5239 participants across 20 villages. Overall scabies prevalence was 15.0% (95%CI 11.8–19.1). There was considerable variation by village with a range of 3.3% to 42.6%. There was a higher prevalence of scabies in males (16.7%) than females (13.5%, adjusted relative risk 1.2, 95%CI 1.1–1.4). Children aged under two years had the highest prevalence (27%). Overall impetigo prevalence was 5.6% (95%CI 4.2–7.3), ranging from 1.4% to 19% by village. The population attributable risk of impetigo associated with scabies was 16.1% (95% CI 9.8–22.4). The prevalence of scabies in our study is comparable to previous studies in Solomon Islands, highlighting a persistent high burden of disease in the country, and the need for public health strategies for disease control.

## Author summary

Scabies is a skin disease caused by infestation with the mite *Sarcoptes scabiei* var. *hominis*. The mite burrows under the skin causing intense itch. Scabies lesions are commonly infected with bacteria, causing impetigo. Pacific Island countries have a high burden of scabies due to overcrowding, the tropical environment and limited access to treatment. We aimed to assess the prevalence of scabies and impetigo in 20 villages in Western Province of Solomon Islands. We used the International Alliance for the Control of Scabies 2020 diagnostic criteria to diagnoses scabies. We found that 15% of the 5239 people surveyed had scabies, and 5.6% had impetigo. While the prevalence of scabies was comparable to other studies there were fewer cases of impetigo than observed previously. The burden of disease was highest among children. Mass drug administration, where the whole community is given treatment for a condition regardless of whether they have symptoms of the disease, has been shown to reduce the prevalence of scabies. This study supports the need for mass drug administration for scabies in Solomon Islands.

## Introduction

Scabies is a skin condition caused by the parasitic mite *Sarcoptes scabiei* var. *hominis*. The mite is primarily transmitted person-to-person by body contact. The mite burrows into the skin causing intense itch and rash via a host immune-mediated response.[1] The host response and scratching can lead to a break in the skin barrier which in turn can lead to secondary bacterial skin infection, most frequently by *Streptococcus pyogenes* and *Staphylococcus aureus*. [2] Superficial bacterial skin infection, known as impetigo, can lead to severe infections and serious immune mediated complications of the internal organs, including the kidney and heart.[3,4]

Scabies is classified by the World Health Organization (WHO) as a neglected tropical disease, with an estimated 455 million annual incident cases.[5] The disease has a high prevalence in many low-resource tropical environments, particularly in Pacific Islands countries.[6] Scabies transmission occurs as a result of skin-to-skin contact and spreads easily in overcrowded living conditions.

Recent surveys in Solomon Islands have found all-age prevalence of scabies in the range 10 to 25% and impetigo 11 to 40%, but the relationship between the two diseases in these studies is not consistent (Table 1). [7–11] For example, a study in 1984 observed a low prevalence of scabies (1.3%) and a high prevalence of impetigo (43%), while a recent survey conducted in school-age children found a high prevalence of both (54.3% and 32.1% respectively).[12,13] The prevalence of scabies and impetigo may have been impacted by living conditions, environmental and demographic factors.

**Table 1:** Scabies surveys in Solomon Islands.

Location	Year study commenced	Sample size	Methodology	Prevalence of scabies % (95%CI)	Prevalence of impetigo % (95%CI)	Population attributable risk impetigo with scabies % (95%CI)
Urban; Western Province (12)	2018	324	1 primary school. Examination of exposed areas by clinician.	54.3% (48.7–59.8)	32.1% (27.0–37.5)	19.2% (2.4–35.9)
Rural; Malaita (9)	2017	118	1 community. Examination by clinician, diagnosis based on IMCI criteria.	10.2% (5.9–16.9)	-	-
Rural; Malaita (8)	2016	1291	6 communities. Examination by clinician.	10.5%	11.2%	-
Rural; Choiseul (7)	2015	1399	10 villages. Examination by study coordinator.	18.7% (16.7–20.8)	24.8% (22.6–27.1)	40.7% (35.0–46.5)
Rural; Western Province (10)	2014	1908	10 villages. Examination by a clinician.	19.2% (16.7–21.9)	26.7% (24.2–29.5)	11.9% (8.6–15.2)
Rural; Malaita (11)	1997	1558	5 islands. Examination in children only.	25% (20–30)	40% (34–47)	-
Rural; Western Province (13)	1984	10,224	48 villages. Examination by two clinicians.	1.3%	43%	-

Mass drug administration (MDA) is an effective control strategy for a number of neglected tropical diseases, including scabies.[14] In high-prevalence settings, two doses of ivermectin-based MDA has been shown to reduce the prevalence of scabies by around 90%.[7,15] The consensus of experts at the WHO Informal Consultation on a Framework for Scabies Control is that public health action such as MDA is recommended where community prevalence of scabies is 10% or greater.[16]

The study was conducted as part of the Regimens of Ivermectin for Scabies Elimination (RISE) trial (ACTRN12618001086257).[17] RISE is a cluster randomised, non-inferiority trial of one versus two doses of ivermectin-based MDA in Solomon Islands. The aim of this study was to determine the baseline prevalence of scabies and impetigo, the relationship between them and their distribution. This study is one of the first to use the 2020 International Alliance for the Control of Scabies (IACS) Criteria for diagnosis of scabies.[18]

## Methods

### Ethics statement

This trial was approved by the Solomon Islands Health Research and Ethics Review Board (HRE005/18) and the Royal Children’s Hospital Human Research Ethics Committee, Melbourne, Australia (38099A). All participants provided written consent to participate. A parent or guardian provided consent for people aged less than 18 years.

### Setting

**Fig 1:**
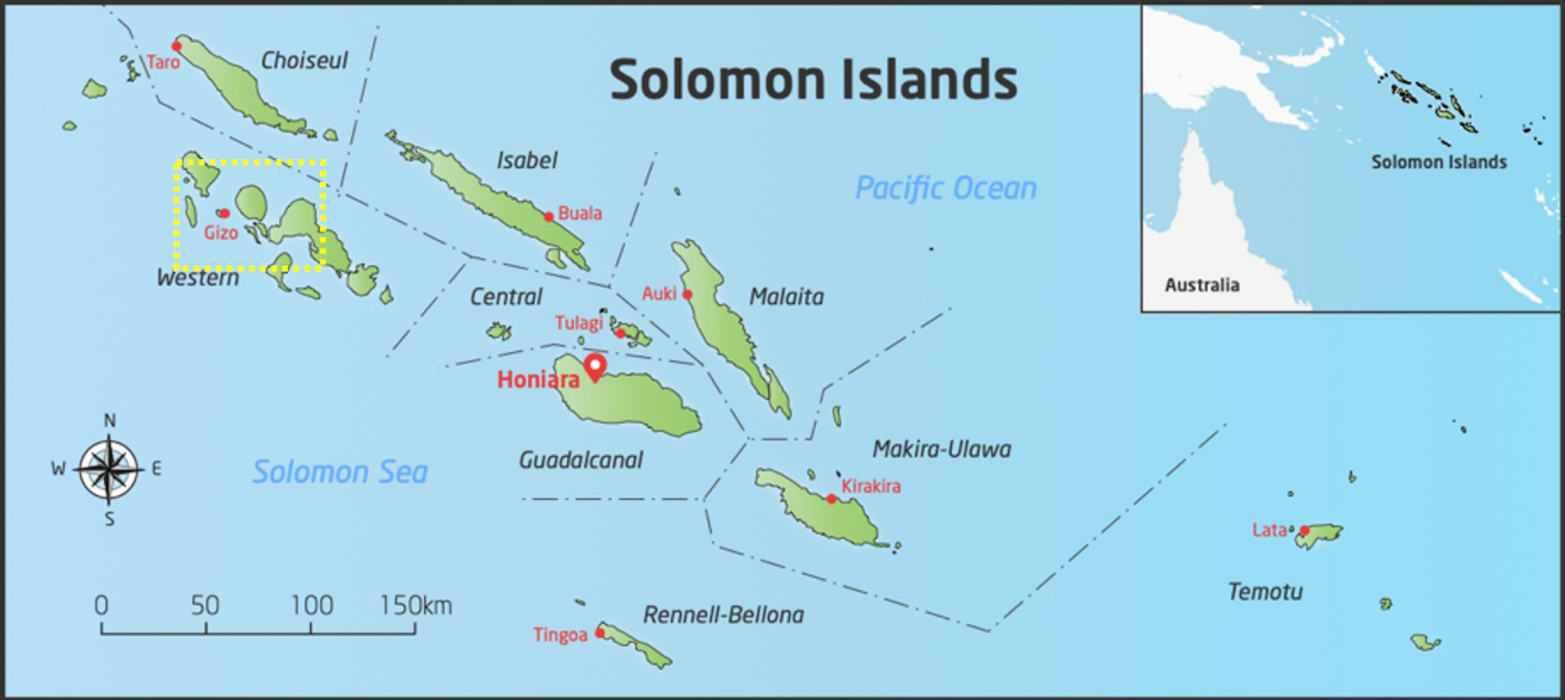
Study area in Western Province, Solomon Islands. **(**The maps in this figure was drawn by Hilary Bruce at Murdoch Children’s Research Institute and adapted by the authors of the manuscript. The source that was used to create the map is freevectormaps.com, https://freevectormaps.com/solomon-islands/SB-EPS-02-0001. **The authors understand and agree to the terms of the Creative Commons Attribution License)**.

We conducted the study in Western Province in Solomon Islands, a country with over 900 islands and a population of approximately 700,000.[19] Solomon Islands is ranked 153 of 189 countries on the Human Development Index.[20] The scattered population presents challenges for health service delivery.[21] Western Province is one of nine provinces of Solomon Islands and has a population of approximately 100,000.[19] It is located in the north-west of the country, close to Papua New Guinea (Fig 1).

Twenty villages were included for the study, each with an estimated population between 200 and 400 people. Villages were selected in close consultation with the Western Provincial Health Services. Criteria for village selection included separation from other villages, population size, and a willingness to participate in the study. The separation of villages from others was considered important for the RISE study so that there was not a high degree of admixing between study villages that received MDA with other villages. Willingness to participate in the study was determined by discussions with the Western Provincial Health Services and consultation with village leaders. Based on information provided by village leaders, the estimated combined population of the 20 villages was approximately 5,500 people. The sample size was calculated for the RISE study, based on previous studies of scabies prevalence in Western Province.[17] Three of the villages had a basic health clinic, while residents of other villages travel for their medical care. The majority of village residents rely on subsistence agriculture for their livelihood, with smaller numbers involved in logging and professional roles.

### Study design

This study was a cross sectional assessment of scabies and impetigo prevalence in each village. Village visits were carried out over an eight-week period between May and July 2019. The study team spent between one and three days in each village. All residents present in the village on the days of the study team’s visit were eligible to participate. There were no exclusion criteria for residents of the study villages to enrol in the study.

### Clinical assessment

Four nurses from Western Province were trained in basic dermatologic clinical assessment (skin examination and history taking), with particular emphasis on the signs and symptoms of scabies as per the 2020 IACS Criteria, and clinical features of impetigo, and how to differentiate these from other skin conditions using clinical criteria, without any specialised equipment.[18] Nurses were taught to refer any suspected cases of crusted scabies for further assessment. The training program included interactive tutorials run over a two-day period conducted by three doctors experienced in scabies and impetigo diagnosis. The nurses then did supervised, practical training at a primary school in Gizo, the largest population centre in the province.[20] The same nurses conducted the clinical assessment in this study. As the training took place approximately 8 months before village visits were conducted, a two-day refresher training program was conducted immediately before village visits commenced. Refresher training consisted of further interactive tutorials, a written test and supervised clinical assessments at the primary school.

Nurses assessed and recorded history and examination features. Participants were also interviewed in regard to history of itch and the presence of itch or typical scabies rash in their household or close contacts. Participants were shown photos of typical scabies rash to help them recognise this pattern among their close contacts. Guardians provided history for infants and young children.

Examination of the skin was limited to easily-exposed body areas (head and neck, hands and arms, legs and feet). A limited examination was deemed appropriate as more widespread examination was not practical in a field setting and examination of hands, feet and lower legs has been shown to detect close to 90% of cases compared to full body examination.[22] Nurses did not examine the breasts, groin or genitals. Examination of children aged less than two years included the trunk, as distribution of scabies may be more widespread in this age group.[18,23]

For each participant, a nurse checked for the presence of scabetic lesions and recorded if these were of typical or atypical appearance for scabies. They also checked for the presence of infected skin lesions (impetigo). Severity of both scabies and impetigo were defined by the number of lesions (very mild 1–2 lesions, mild 3–10 lesions, moderate 11–50 lesions, severe >50 lesions). All data were entered directly into a database using Android devices in the field. Study data were collected and managed using REDCap electronic data capture tools hosted at Murdoch Children’s Research Institute.[24,25] REDCap is a secure, web-based software platform designed to support data capture for research studies.

Impetigo was defined as papular, pustular or ulcerative lesions surrounded by erythema, or with crusts, pus or bullae.[26] Severe infections including abscesses, ulcers, cellulitis and crusted scabies were also recorded. Abscesses, cellulitis and secondarily infected scabies lesions were classified as impetigo for data analysis.

### Statistical analysis

Using the data from skin examinations and history features recorded by the nurses, we made a scabies diagnosis for each individual according to the IACS criteria. When calculating the prevalence of scabies, we defined scabies as per the 2020 IACS Criteria subcategories B3 (clinical scabies), C1 and C2 (suspected scabies), which utilise both history and clinical features.[18] We calculated the prevalence of scabies and impetigo overall, as well as for demographic groups. We analysed scabies and impetigo prevalence across ten age groups to provide detailed information of scabies and impetigo prevalence. We then used three broader age groups (infants [under 2 years], children [2 to 14 years], and adults [15 years and above]) for further analysis of diagnostic criteria’. A generalised linear model with a log link and binomial distribution was used to model scabies and impetigo, adjusted for clustering by village. This model was used to calculate confidence intervals (CI) for scabies and impetigo prevalence and adjusted relative risk (ARR) for each category. The age group of those aged 50–59 years was used as the reference group for calculating ARR for scabies and impetigo. We calculated population attributable risk as a measure of the effect on population incidence of impetigo if scabies was eliminated. We also calculated the Pearson correlation coefficient of scabies and impetigo prevalence by village. All data analysis was performed using Stata (version 14.2, StataCorp, College Station, TX, USA).

## Results

We enrolled 5,239 participants, representing more than 95% of the estimated population of the study villages (Tables 2 and S1). The enrolled population had comparable age and sex structure to the overall population of Western Province in the 2009 national census but there were some differences (S1 Fig).[27] There was a higher proportion of people aged less than 15 years in the study sample compared to the census (48% vs. 40%), and a higher proportion of females (52.7% vs. 47.9%); in particular there were fewer males aged 20–39 years in the study. Enrolment per village ranged from 109 to 443 participants (S2 Table). Enrolment in some villages was higher than the estimated population because of visitors who were staying in the study village at the time.

**Table 2:** Prevalence of scabies and impetigo adjusted for village clustering.

**Study sample**			**Scabies**			**Impetigo**		
	N (%)		n	% (95%CI)	ARR	n	% (95%CI)	ARR
**Sex**								
**Female**	2762 (52.7)		374	13.5 (0.6–17.2)	Ref	131	4.7 (3.4–6.5)	Ref
**Male**	2477 (47.3)		413	16.7 (13.0–21.4)	1.2 (1.1–1.4)	160	6.5 (5.0–8.4)	1.4 (1.1–1.7)
**Age (years)**								
**0–1**	263 (5.0)		71	27.0 (21.1–34.5)	3.1 (2.2–4.3)	18	6.8 (4.0–11.7)	5.9 (1.8–19.5)
**2–4**	542 (10.3)		122	22.5 (17.1–29.7)	2.6 (1.7–4.0)	60	11.1 (7.2–17.0)	9.5 (2.9–30.5)
**5–9**	904 (17.3)		162	17.9 (13.4–23.9)	2.0 (1.2–3.4)	81	9.0 (6.2–13.0)	7.7 (3.0–19.9)
**10–14**	806 (15.4)		139	17.2 (12.8–23.2)	2.0 (1.3–2.9)	62	7.7 (5.6–10.6)	6.6 (2.4–17.7)
**15–19**	383 (7.3)		40	10.4 (7.1–15.3)	1.2 (0.8–1.8)	20	5.2 (3.6–7.7)	4.5 (1.3–15.7)
**20–29**	609 (11.6)		69	11.3 (7.8–16.5)	1.3 (0.9–1.8)	13	2.1 (1.2–3.8)	1.8 (0.5–6.7)
**30–39**	608 (11.6)		72	11.8 (8.5–16.5)	1.4 (0.9–2.0)	14	2.3 (1.5–3.6)	2.0 (0.6–6.2)
**40–49**	446 (8.5)		43	9.6 (6.3–14.7)	1.1 (0.7–1.8)	11	2.5 (1.5–4.1)	2.1 (0.7–6.1)
**50–59**	342 (6.5)		30	8.8 (5.5–14.0)	Ref	4	1.2 (0.4–3.3)	Ref
**60+**	336 (6.4)		39	11.6 (7.2–18.8)	1.3 (0.8–2.2)	8	2.4 (1.0–5.5)	2.0 (0.6–6.9)
**Total**	5239		787	15.0 (11.8–19.1)		291	5.6 (4.2–7.3)	

(CI: confidence interval; ARR: relative risk)

### Scabies prevalence

There were 787 participants who met the diagnostic criteria for scabies (prevalence 15%, 95%CI 11.8–19.1). The prevalence was higher in males than females (16.7% vs. 13.5%, ARR 1.2, 95%CI 1.1–1.4) and in children aged less than 2 years (27%, ARR 3.1, 95%CI 2.2–4.3 compared to those aged 50 to 59 years, Table 2).

**Fig 2:**
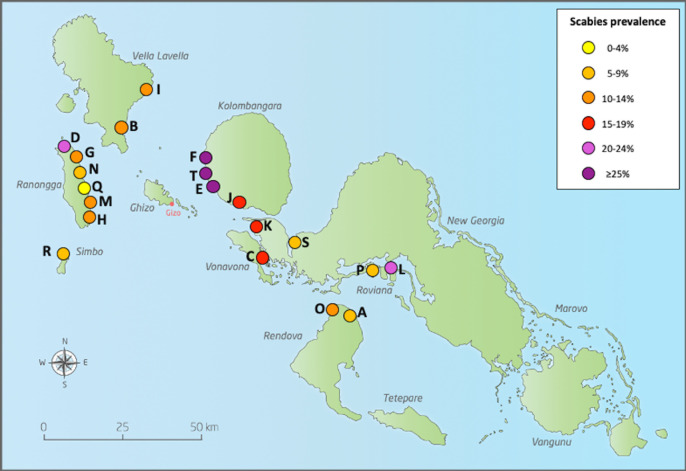
Geographical distribution of scabies in Western Province, Solomon Islands. (The maps in this figure was drawn by Hilary Bruce at Murdoch Children’s Research Institute and adapted by the authors of the manuscript. The source that was used to create the map is freevectormaps.com, https://freevectormaps.com/solomon-islands/SB-EPS-02-0001. **The authors understand and agree to the terms of the Creative Commons Attribution License)**.

Scabies prevalence varied by village (Fig 2 and S2 Table). The median village prevalence was 12.5% (IQR 8.9 to 20.0) with a range of 3.3% to 42.6%. The prevalence was higher than the survey median in all villages on the island of Kolombangara (villages E, F, J, T) with median 25.7% and range 19.5 to 42.6%.

Overall, 51% of participants with scabies had very mild or mild disease (<10 lesions); and 15.3% had severe disease (>50 lesions, Table 3). No cases of crusted scabies were recorded. Among participants with scabies, participants aged less than 2 years had the highest proportion of moderate or severe scabies (71.8%, 95%CI 59.9–81.9), twice that of those aged 15 years and above (35.8%, 95%CI 41.6). Nearly all participants diagnosed with scabies had lesions on their upper limbs (96.2%), while 44.7% had scabies lesions on their lower limbs (Table 4).

**Table 3:** Diagnosis and severity of scabies and impetigo.

	Total	Age			Sex	
	N (%)	<2 years n (%)	2–14 years n (%)	≥ 15 years n (%)	Male n (%)	Female n (%)
***Scabies***						
**Prevalence**	787 (15.0)	71 (27.0)	423 (18.8)	293 (10.8)	413 (16.7)	374 (13.5)
**2020 IACS Category**						
B3: Typical lesions in a typical distribution and 2 history features	439 (55.8)	47 (66.2)	260 (61.5)	132 (45.1)	233 (56.4)	206 (55.1)
C1: Typical lesions in a typical distribution and 1 history feature	205 (26.0)	21 (29.6)	114 (27.0)	70 (23.9)	115 (27.8)	90 (24.1)
C2: Atypical lesions or atypical distribution and 2 history features	143 (18.2)	3 (4.2)	49 (11.6)	91 (31.1)	65 (15.7)	78 (20.9)
**Severity**						
Very mild (1–2 lesions)	37 (4.7)	0	11 (2.6)	26 (8.9)	15 (3.6)	22 (5.9)
Mild (3–10 lesions)	364 (46.3)	20 (28.2)	182 (43.0)	162 (55.3)	182 (44.1)	182 (48.7)
Moderate (11–50 lesions)	266 (33.8)	28 (39.4)	162 (38.3)	76 (25.9)	149 (36.1)	117 (31.3)
Severe (>50 lesions)	120 (15.3)	23 (32.4)	68 (16.1)	29 (9.9)	67 (16.2)	53 (14.2)
***Impetigo***						
**Prevalence**	291 (5.6)	18 (6.8)	203 (9.0)	70 (2.6)	160 (6.5)	131 (4.7)
**Severity**						
Very mild (1–2 lesions)	196 (67.4)	4 (22.2)	136 (67.0)	56 (80.0)	99 (61.9)	97 (74.0)
Mild (3–10 lesions)	79 (27.1)	12 (66.7)	55 (27.1)	12 (17.1)	51 (31.9)	28 (21.4)
Moderate (11–50 lesions)	9 (3.1)	2 (11.1)	7 (3.4)	0	5 (3.1)	4 (3.1)
Severe (>50 lesions)	3 (1.0)	0	3 (1.5)	0	3 (1.9)	0
Unknown	4 (1.4)	0	2 (1.0)	2 (2.9)	2 (1.3)	2 (1.5)
**Total**	5239	263	2252	2724	2477	2762

**Table 4:** Scabies diagnosis by 2020 IACS Criteria.

	Total	Positive scabies diagnosis	Negative scabies diagnosis	Age			Sex	
	N (%)	Present n (%)	Present n (%)	<2 years n (%)	2–14 years n (%)	≥ 15 years n (%)	Male n (%)	Female n (%)
**Examination findings**								
Typical scabies lesions	793 (15.1)	645 (82.0)	148 (3.3)	83 (31.6)	478 (21.2)	232 (8.5)	430 (17.4)	363 (13.1)
Atypical scabies lesions	497 (9.5)	142 (18.0)	355 (8.0)	29 (11.0)	235 (10.4)	233 (8.6)	241 (9.7)	256 (9.3)
**Distribution of scabies lesions**								
Upper limbs	1229 (23.5)	757 (96.2)	472 (10.6)	101 (38.4)	685 (30.4)	443 (16.3)	643 (26.0)	586 (21.2)
Lower limbs	469 (9.0)	352 (44.7)	117 (2.6)	84 (31.9)	264 (11.7)	121 (4.4)	243 (9.8)	226 (8.2)
Head	43 (0.8)	36 (4.6)	7 (0.2)	13 (4.9)	23 (1.0)	7 (0.3)	24 (1.0)	19 (0.7)
Trunk*	43 (0.8)	38 (4.8)	5 (0.1)	8 (3.0)	29 (1.3)	6 (0.2)	19 (0.8)	24 (0.9)
Lesions in any location	1289 (24.6)	787 (100)	502 (11.3)	112 (42.6)	712 (31.6)	465 (17.1)	671 (27.1)	618 (22.4)
**History features**								
***Itch***								
Itch present	1254 (23.9)	706 (83.8)	548 (12.5)	71 (27.0)	531 (23.6)	652 (23.9)	634 (25.6)	620 (22.4)
***Contact history***								
Any positive contact history	1522 (29.1)	663 (84.2)	859 (19.3)	88 (33.5)	664 (29.5)	770 (28.3)	731 (29.5)	791 (28.6)
Household contact w/itch	1070 (20.4)	574 (72.9)	496 (11.1)	68 (25.9)	486 (21.6)	516 (18.9)	520 (21.0)	550 (19.9)
Close contact w/itch	687 (13.1)	335 (42.6)	352 (7.9)	42 (16.0)	296 (13.1)	349 (12.8)	314 (12.7)	373 (13.5)
Household contact w/typical scabies lesions	882 (16.8)	493 (62.6)	389 (8.7)	59 (22.4)	399 (17.7)	424 (15.6)	421 (17.0)	461 (16.7)
Close contact w/typical scabies lesions	792 (15.1)	323 (41.0)	469 (10.5)	48 (18.3)	351 (15.6)	393 (14.4)	370 (14.9)	422 (15.3)
**Total**	5239	787	4452	263	2252	2724	2477	2762

* Trunk not routinely examined in participants ≥2 years old

### Scabies diagnosis by 2020 IACS criteria

Of those with scabies, 439 (55.8%) had clinical scabies (subcategory B3) and 348 (44.2%) had suspected scabies (subcategories C1: 26% and C2: 18.2%,) according to the 2020 IACS Criteria classifications (Table 4). Among participants with scabies aged less than 2 years, 66.2% were in subcategory B3, while among those aged 2 to 14 years 61.5% were in B3 and 45.1% in participants aged 15 years and older. The subcategorization between males and females was similar. There were 503 participants (9.6%) with documented skin lesions on examination (148 with typical scabies lesions, 355 with atypical scabies lesions) that did not meet the 2020 IACS criteria for scabies diagnosis due to a lack of history features (presence of itch or positive contact history).

On history, 23.9% of all participants reported itch. This was significantly higher in participants with scabies (83.8%) than in those without scabies (12.5%, ARR 7.3, 95%CI 6.7–7.9). Overall prevalence of itch was similar across ages and by sex. Overall, 29.1% had a positive contact history. This was higher in those with scabies (84.2%) compared to those without scabies (19.3%, ARR 4.4, 95%CI 4.1–4.7). Children under the age of two years were most likely to have positive contact history (33.5%) although the differences across age groups were not significant. The most sensitive contact history feature was reporting a household contact with itch (72.9% of participants with scabies). The least sensitive contact history feature was reporting a close contact with typical scabies lesions (41% of participants with scabies). The contact history from close contacts reported in children was similar to adults despite children having many close contacts in school.

### Impetigo prevalence

Impetigo was diagnosed in 291 participants (5.6%, 95%CI 4.2–7.3). Prevalence was higher among males (6.5% vs. 4.7% in females, ARR 1.4, 95%CI 1.1–1.7), and highest among children aged 2 to 4 years (11.1%, ARR 9.5, 95%CI 2.9–30.5 compared to those aged 50–59 years, Table 2). Among participants with impetigo, the majority had very mild disease (≤2 lesions, 67.4%, Table 3). There were 12 participants (4.1%) with moderate to severe impetigo (>11 lesions), all aged less than 15 years, and half of them aged between 1 and 3 years.

**Fig 3:**
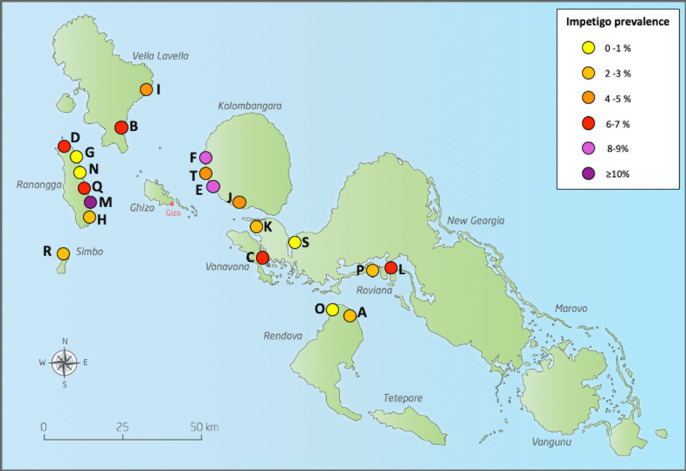
Geographical distribution of impetigo in Western Province, Solomon Islands. (The maps in this figure was drawn by Hilary Bruce at Murdoch Children’s Research Institute and adapted by the authors of the manuscript. The source that was used to create the map is freevectormaps.com, https://freevectormaps.com/solomon-islands/SB-EPS-02-0001. **The authors understand and agree to the terms of the Creative Commons Attribution License)**.

When analysed by village, the median village impetigo prevalence was 5.1% (IQR 2.6 to 6.8, range 1.4% to 19%). Unlike scabies, there was no distinct pattern in the geographical distribution of impetigo (Fig 3).

### Association between scabies and impetigo

Of the participants with scabies, 81 of 787 (10.3%) had impetigo, a higher proportion than participants without scabies (210 of 4452, 4.6%; RR 2.2, 95%CI 1.8–2.7). The population attributable risk of impetigo associated with scabies was 16.1% (95%CI 9.8–22.4). The Pearson correlation coefficient of the relationship between scabies and impetigo by village was 0.28 (*P* = 0.24), indicating a weak positive relationship that was not statistically significant (S2 Fig).

## Discussion

We observed a very high prevalence of scabies (15.0%) and a moderately high prevalence of impetigo (5.6%) in our study of over 5,000 participants of all ages in Solomon Islands. Young children were especially affected by both conditions. These findings highlight the ongoing high burden of scabies in the country and provide further impetus for public health action to curb the impact of the disease.

The epidemiology of scabies has been studied in three of the nine provinces of Solomon Islands (Table 1). With the exception of an early 1984 study, all studies observed a high prevalence of scabies. Our findings were comparable to a 2014 study in Western Province (prevalence 19.2%)[10] Our study included three of the 10 villages sampled in the 2014 study.

While still considered to be a moderately high prevalence of impetigo, the prevalence we observed (5.6%) was lower than the 2014 study (32.7%) and other studies in Solomon Islands (prevalence range 11.2% - 43%). The lower prevalence may reflect a real change, possibly due to social, environmental factors and health care system factors. Our survey was conducted at the beginning of the dry season, while the 2014 survey was conducted during the wet season, when bacterial skin infections are more likely to occur.[10,28] It may also be that our study under-diagnosed impetigo. Clinical assessments in our study were made by nurses who had completed a brief training package. In a study of the accuracy of this approach, nurses had a sensitivity of 53% for diagnosis of impetigo compared to expert physicians, and were particularly likely to miss mild disease.[20] The diagnosis of scabies had a slightly similar sensitivity of 55%. Given these findings, we ran further training for nurses prior to starting our study, particularly focussing on detecting mild cases. However, under-diagnosis may still have occurred.

There was substantial variation between villages in prevalence of scabies (range 3 to 43%) and impetigo (1 to 19%). We observed a weak relationship between scabies and impetigo prevalence by village which was not statistically significant. The variation may be partly explained by differences in geography and climate. For example, on Kolombangara island, which experiences a high amount of rainfall and cooler temperatures than other islands, all villages had a scabies prevalence of over 19%. While some studies have shown a positive correlation between rainfall or cold weather and the incidence of scabies, others have shown no increase in scabies prevalence in the wet season.[28–30] While it is known that crowding can influence scabies prevalence and transmission we did not collect information on living conditions.[1] Despite the pathophysiological link between scabies and impetigo, a 2015 systematic review of scabies and impetigo prevalence worldwide did not find a strong correlation between the prevalence of the two diseases across populations.[6] The relationship between the two diseases may differ over time and by place, due to local factors that are not well understood. The differences in observed prevalence may be explained by natural fluctuation, differences in the diagnostic methods or other, unidentified factors.[31]

This study had several limitations. First, the history components of the clinical assessment may have been under-reported due to normalisation of itch and skin lesions in communities.[32] In a setting where one in six people have scabies, it is likely that most people would have a close contact with scabies, however less than 30% reported a positive contact history. Second, while there were advantages to using trained nurses to conduct the clinical assessment, this may have resulted in reduced diagnostic accuracy of both scabies and impetigo compared to specialists. Mild cases may have been under-diagnosed, as found previously.[20] Third, the study population represents approximately 5% of the population of Western Province. While there was a high level of participation in the study villages there are some differences in the age and sex of the study population compared to the population of Western Province. There is a degree of bias in the selection of study villages as they were required to be a certain size for the RISE study and therefore did not capture very small villages or urban populations.

There is an ongoing, high prevalence of scabies and impetigo in Solomon Islands. Further studies in other provinces and urban settings would help to understand the national burden of the disease. The methodology used in this study may be appropriate for future scabies and impetigo prevalence studies. The limited skin examination is appropriate for a field setting. Diagnosis of scabies according to the 2020 IACS Criteria allows direct comparison between the results of this study and others that have used the same diagnostic criteria. Public health strategies are needed, with parallel operational research to evaluate the effectiveness of these strategies. Studies, such as the RISE trial, into community control of scabies will help to inform policy to eliminate scabies as a public health problem.

## Supporting information

S1 Table Participant demographics. (DOCX)

S2 Table Scabies and impetigo prevalence in villages. (DOCX)

S1 Fig Population pyramid of census data for study population and study sample. (TIFF)

S2 Fig Correlation of scabies and impetigo prevalence in villages. Pearson correlation coefficient r = 0.28 (P = 0.24)
(TIFF)
