# Supplementary material for: Defining the need for public health control of scabies in Solomon Islands
Source: PLoS Negl Trop Dis. 2021 Feb 22;15(2):e0009142. doi: 10.1371/journal.pntd.0009142 (PMC7932527; doi:10.1371/journal.pntd.0009142)
Supplement: S1 Table — (DOCX) [file pntd.0009142.s001.docx]

## Table S1. Participant demographics

| **Age group (years)** | **Total** | | **Male** | | **Female** | |
| --- | --- | --- | --- | --- | --- | --- |
|  | **N** | **%** | **n** | **%** | **n** | **%** |
| 0-1 | 263 | 5.0 | 128 | 48.7 | 135 | 51.3 |
| 2-4 | 542 | 10.4 | 272 | 50.2 | 270 | 49.8 |
| 5-9 | 904 | 17.3 | 463 | 51.2 | 441 | 48.8 |
| 10-14 | 806 | 15.4 | 417 | 51.7 | 389 | 48.3 |
| 15-19 | 383 | 7.3 | 180 | 47.0 | 203 | 53.0 |
| 20-29 | 609 | 11.6 | 227 | 37.3 | 382 | 62.7 |
| 30-39 | 608 | 11.6 | 242 | 39.8 | 366 | 60.2 |
| 40-49 | 446 | 8.5 | 222 | 49.8 | 224 | 50.2 |
| 50-59 | 342 | 6.5 | 161 | 47.1 | 181 | 52.9 |
| 60+ | 336 | 6.4 | 165 | 49.1 | 171 | 50.9 |
| **Total** | 5239 | 100 | 2477 | 47.3 | 2762 | 52.7 |
