# Supplementary material for: Defining the need for public health control of scabies in Solomon Islands
Source: PLoS Negl Trop Dis. 2021 Feb 22;15(2):e0009142. doi: 10.1371/journal.pntd.0009142 (PMC7932527; doi:10.1371/journal.pntd.0009142)
Supplement: S2 Table — (DOCX) [file pntd.0009142.s002.docx]

## Table S2. Scabies and impetigo prevalence in villages

| **Village** | **Study sample** | **Scabies prevalence**  **N (%, 95% CI)** | **Impetigo prevalence**  **N (%, 95% CI)** |
| --- | --- | --- | --- |
| A | 258 | 22 (8.5, 5.4-12.6) | 9 (3.5, 1.6-6.5) |
| B | 443 | 53 (12.0, 9.1-15.4) | 29 (6.5, 4.4-9.3) |
| C | 372 | 67 (18.0, 14.2-22.3) | 25 (6.7, 4.4-9.8) |
| D | 345 | 71 (20.6, 16.4-25.2) | 21 (6.1, 3.8-9.2) |
| E | 190 | 81 (42.6, 35.5-50.0) | 17 (8.9, 5.3-13.9) |
| F | 290 | 75 (25.9, 20.9-31.3) | 26 (9.0, 5.9-12.9) |
| G | 281 | 30 (10.7, 7.3-14.9) | 4 (1.4, 0.4-3.6) |
| H | 279 | 38 (13.6, 9.8-18.2) | 6 (2.2, 0.8-4.6) |
| I | 202 | 26 (12.9, 8.6-18.3) | 11 (5.4, 2.7-9.5) |
| J | 298 | 58 (19.5, 15.1-24.4) | 14 (4.7, 2.6-7.8) |
| K | 266 | 43 (16.2, 12.0-21.2) | 10 (3.8, 1.8-6.8) |
| L | 191 | 39 (20.4, 14.9-26.8) | 13 (6.8, 3.7-11.4) |
| M | 211 | 22 (10.4, 6.7-15.4) | 40 (19.0, 13.9-24.9) |
| N | 268 | 22 (8.2, 5.2-12.2) | 5 (1.9, 0.6-4.3) |
| O | 183 | 19 (10.4, 6.4-15.7) | 3 (1.6, 0.3-4.7) |
| P | 177 | 15 (8.5, 4.8-13.6) | 6 (3.4, 1.3-7.2) |
| Q | 338 | 11 (3.3, 1.6-5.7) | 26 (7.7, 5.1-11.1) |
| R | 264 | 15 (5.7, 3.2-9.2) | 8 (3.0, 1.3-5.9) |
| S | 109 | 10 (9.2, 4.5-16.2) | 2 (1.8, 0.2-6.5) |
| T | 274 | 70 (25.6, 20.5-31.1) | 16 (5.8, 3.4-9.3) |

## 
