# Supplementary figures and images for: Defining the need for public health control of scabies in Solomon Islands
Source: PLoS Negl Trop Dis. 2021 Feb 22;15(2):e0009142. doi: 10.1371/journal.pntd.0009142 (PMC7932527; doi:10.1371/journal.pntd.0009142)

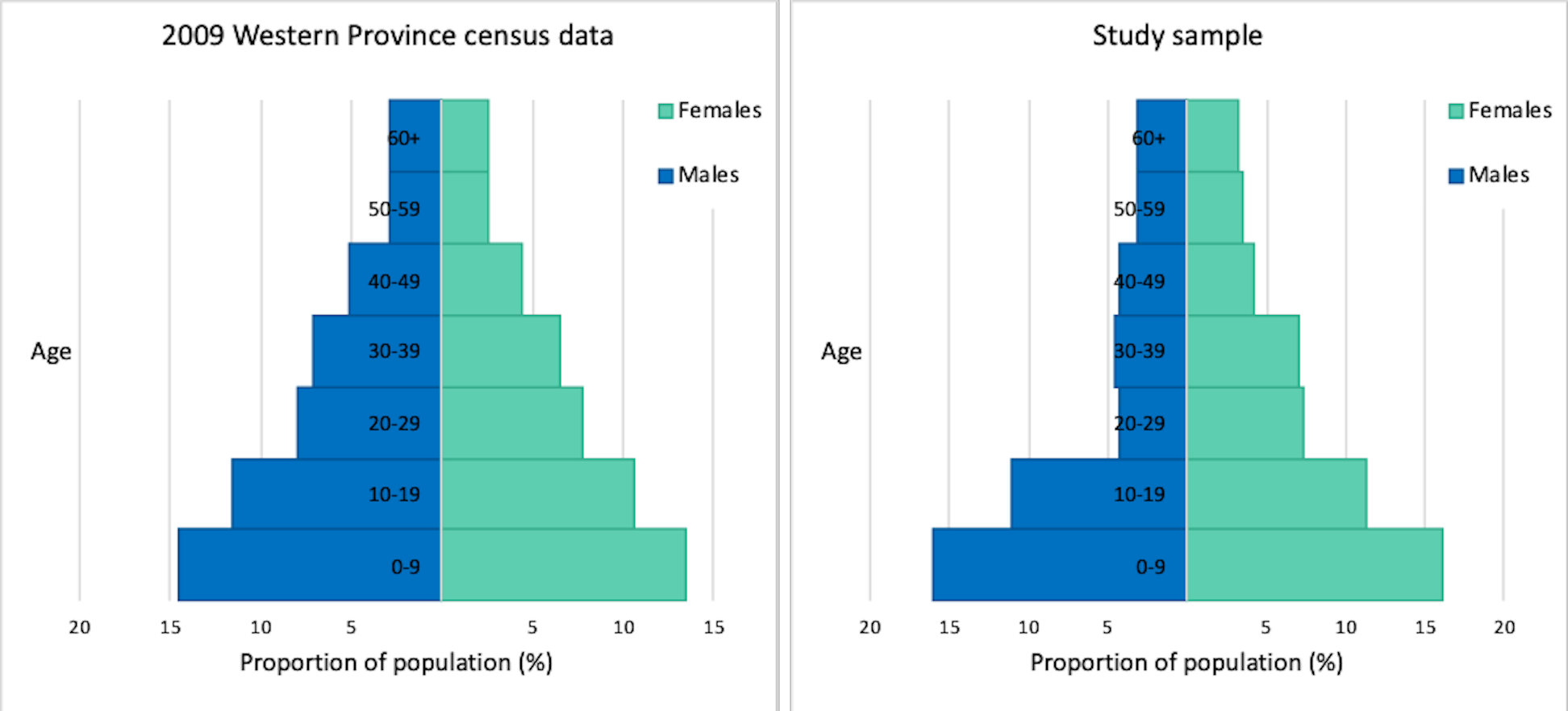

Supplement: S1 Fig — (TIFF) [file pntd.0009142.s003.tiff]

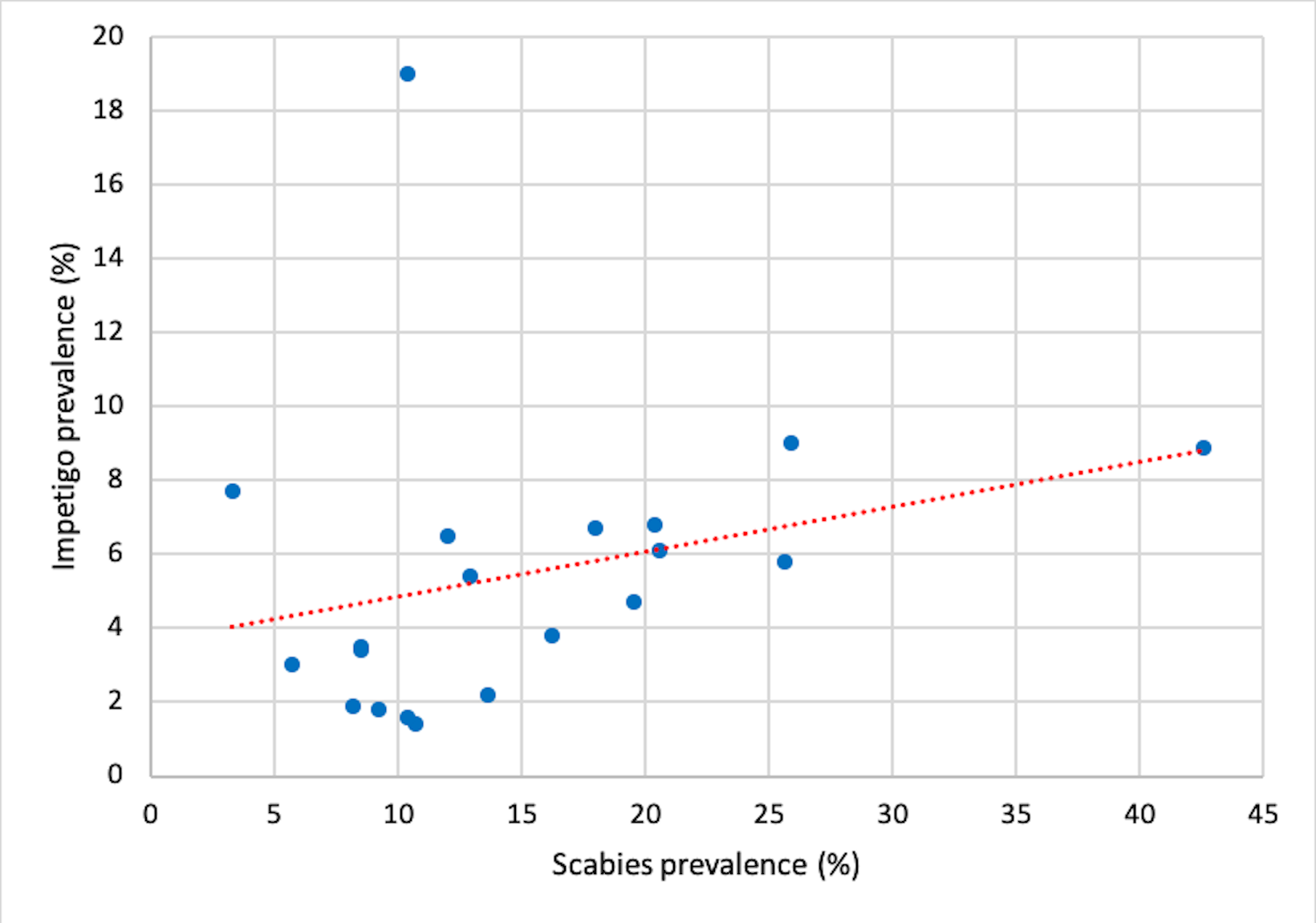

Supplement: S2 Fig — Pearson correlation coefficient r = 0.28 (P = 0.24) (TIFF) [file pntd.0009142.s004.tiff]
